# Utility of diffusion tensor imaging for assessing bone quality in type 2 diabetes: correlation with bone turnover biomarkers

**DOI:** 10.1007/s11657-025-01643-z

**Published:** 2026-01-12

**Authors:** Amany A. Mousa, Alaa M. Wafa, Randa Ramadan, Rasha Elzehery, Nehal Tharwat

**Affiliations:** 1 Department of Internal Medicine, Endocrine, Diabetes and Clinical Nutrition Unit, Faculty of Medicine, Specialized Medical Hospital, Mansoura University, Mansoura, Egypt; 2 Department of Clinical Pathology, Faculty of Medicine, Mansoura University, Mansoura, Egypt; 3 Department of Diagnostic Radiology, Faculty of Medicine, Mansoura University, Mansoura, Egypt

**Keywords:** Type 2 diabetes mellitus, Bone quality, Diffusion tensor imaging, Fractional anisotropy, Bone turnover markers

## Abstract

***Summary*:**

We examined type 2 diabetic patients by diffusion tensor imaging, an advanced magnetic resonance technique, and found significant differences than healthy subjects that correlated with markers of bone turnover. This could help in assessment of bone quality in diabetics where bone density often appears normal despite high fracture risk.

**Introduction:**

Type 2 diabetes (T2DM) induced bone fragility is mainly due to alteration of bone quality rather than a decline in bone mineral density. The aim of the present study was to assess the ability of diffusion tensor imaging (DTI) to assess bone quality in T2DM.

**Methods:**

A total of 68 patients with T2DM were enrolled and stratified into two groups: those with microvascular complications (*n* = 38) and those without (*n* = 30). Additionally, 30 age- and sex-matched healthy controls were included. All participants underwent DTI of the lumbosacral spines and both hips, dual-energy X-ray absorptiometry (DEXA) scanning, and assessment of bone turnover biomarkers, including C-terminal telopeptide of type I collagen (CTX) and procollagen type I N-terminal propeptide (P1NP).

**Results:**

DTI parameters—fractional anisotropy (FA) and apparent diffusion coefficient (ADC)—differed between patients and controls. FA values for the spine and hips were lower, while ADC values for the hips were higher in both diabetic patient groups compared to control subjects. ADC values for the spine were higher in diabetic patients with complications than in controls. A significant positive correlation was observed between FA and CTX levels in patients with complications. No significant differences were found in DEXA scan results between patients and controls. FA and ADC values for the hips showed the highest area under the curve (AUC) values (0.868 and 0.818, respectively), followed by FA and ADC for the spine (0.779 and 0.721), according to ROC curve analysis. The optimal cutoffs yielding the best sensitivity and specificity were observed for FA over ADC.

**Conclusion:**

DTI can provide information about the microstructural changes associated with the development of a low bone turnover state in type 2 diabetes mellitus, where bone mineral density typically remains within the normal range.

## Introduction

Diabetes is associated with an increased risk of fractures despite normal or even elevated bone mineral density (BMD) [1]. Studies have shown that the risk of hip fracture is 1.4 times higher in patients with type 2 diabetes mellitus (T2DM) compared to non-diabetic individuals [2]. A vertebral fracture prevalence of 37–50% has been reported in diabetic populations [3]. Patients with T2DM have a higher likelihood of fractures regardless of BMD, suggesting that the primary mechanism underlying T2DM-induced bone fragility may be related to alterations in bone quality rather than a reduction in BMD [4]. Research has also indicated that individuals with T2DM exhibit reduced bone turnover, particularly in markers of bone formation such as osteocalcin (OC) and procollagen type I N-terminal peptide (P1NP), as well as in the bone resorption marker C-terminal telopeptide of type I collagen (CTX). CTX levels have been found to be negatively correlated with metabolic control. Evidence suggests that decreased bone turnover may impair the repair of microcracks in patients with T2DM, contributing to skeletal fragility [5].

T2DM is also associated with compromised skeletal microarchitecture. Patients often exhibit reduced trabecular thickness, increased porosity, and cortical thinning, all of which contribute to weakened cancellous bone and reduced breaking strength [6, 7]. Dual-energy X-ray absorptiometry (DXA) has limited ability to predict fracture risk, likely due to its inability to capture the full spectrum of cancellous bone characteristics, as it assesses only the mineral component. Additional factors influencing bone strength and fracture resistance include the topological properties of the trabecular network, bone turnover, and bone marrow composition [8]. In this context, unlike DXA, magnetic resonance (MR) techniques can provide insights into and bone marrow composition [9]. Diffusion tensor imaging (DTI) is an advanced MR technique that captures signals based on the movement of water molecules [10]. DTI-derived parameters, such as fractional anisotropy (FA) and apparent diffusion coefficient (ADC), have been proposed as indicators of bone microstructure [11]. The aim of the present study was to assess the utility of diffusion tensor imaging, in combination with bone turnover markers CTX and P1NP, for evaluating bone quality in patients with T2DM.

### Methods

This case–control study was conducted at the Endocrinology Outpatient Clinic of Mansoura Specialized Medical Hospital and Diagnostic Radiology Department, Mansoura University, between 2020 and 2023. Patients were consecutively recruited during this period. Written informed consent was obtained from all participants. The study protocol was approved by the Institutional Research Board (IRB) and the local ethics committee at Mansoura University in December 2020, code no. MD.20.12.393. The study included 68 adult patients with type 2 diabetes mellitus (T2DM), comprising premenopausal females and males aged ≤ 50 years. Patients were divided into two groups: 38 with microvascular complications and 30 without. Additionally, 30 healthy individuals, matched for age, sex, and body mass index (BMI), were included as the control group. Diabetic patients with microvascular complications were identified based on standard criteria, including evidence of retinopathy on ophthalmologic examination, nephropathy defined by persistent albuminuria (≥ 30 mg/g creatinine) or eGFR < 60 mL/min/1.73 m^2^, and neuropathy confirmed by clinical assessment (sensory symptoms with monofilament testing or nerve conduction studies). Exclusion criteria were as follows: 1) Thyroid and parathyroid disorders, hepatic or renal failure; 2) Connective tissue disorders, malignancy, and smoking. 3) Patients taking drugs such as steroids, anticoagulants, and pioglitazone; 4) Female receiving hormonal contraception,, pregnancy or with previous history of oophorectomy; 5) Previous joint replacements or fractures during last year; 6)Contraindications for MRI acquisition, such as cardiac pacemaker implants.

#### Laboratory assessment

The determination of the biochemical markers of bone turnover required twelve hours of fasting before obtaining the samples between 8 and 10 a.m. with minimal stasis to avoid hemolysis. Samples were centrifuged and frozen immediately after extraction***.*** Collection of samples and storage: The blood samples were aseptically collected, centrifuged to get serum samples to be aliquoted, and stored at ≤ -20 °C till the time of the assay [12].

A double-antibody sandwich enzyme-linked immunosorbent assay (ELISA) kit was used to assay the level of Human cross-linked C-telopeptide of type I collagen (CTX) in samples. The kit was supplied by Shanghai Sunred Biological Technology Co., Ltd. (Shanghai, China; Catalogue No. 201–12-1495, eMail:sunredbio@msn.cn). Sensitivity of the kit:0.543 ng/ml.

A double-antibody sandwich enzyme-linked immunosorbent assay (ELISA) kit was used to assay the level of Human procollagen I N-terminal peptide (PINP) in samples. The kit was supplied by Shanghai Sunred Biological Technology Co., Ltd. (Shanghai, China; Catalogue No. 201–12-1351, eMail:sunredbio@msn.cn). Sensitivity of the kit: 5.125 ng/ml.

Intra-assay Precision: 3 samples with low, middle and high level human PINP were tested 20 times on one plate, respectively. Inter-assay Precision: 3 samples with low, middle and high level human PINP were tested on 3 different plates, 8 replicates in each plate.

Coefficient of variation (CV) % = SD/meanX100 Intra-Assay: CV < 10%,Inter-Assay: CV < 12%.

**DXA scan:** DXA have been carried out by GE-Lunar prodigy pro version.

#### MR imaging for assessment of bone quality

MR imaging was performed using a 1.5 T magnet (Siemens Healthineers model name Aera serial numer142230) using a lumbar and hip coils. The protocol included: conventional sequences (axial T1 & T2, sagittal T1, T2 and STIR for lumbar spine), (axial T1, T2, STIR and coronal STIR for both hip joints), DTI was performed in sagittal plane for lumbosacral spines and coronal plane for both hips.

#### Diffusion tensor MRI

A single-shot EPI sequence with parallel imaging and automatic multi-angle-projection shimming was used to provide DT imaging. Implementation of diffusion gradients along 32 axes was done using DW factors (b-value) of 0 and 1000 s/mm2. The scanning parameters were as follows:

**For Spines;** TR/TE = 3700/104 ms, FOV = 262 mm, Number of averages: 14, The data matrix is 92 × 88, Voxel dimensions: 2.43 × 2.54 × 2.5 mm3. Forty-eight slices were obtained with a slice thickness of 2.5 mm and no inter-slice gap. The total examination time was 4.5 min. **For Hips;** TR/TE = 4900/104 ms. FOV = 376 mm; number of averages = 14. The data matrix is 92 × 88.Voxel dimensions: 2.43 × 2.54 × 2.5 mm3. Forty-eight slices were obtained with a slice thickness of 3 mm and no inter-slice gap.The total examination time was 4.5 min.

A measurement region as lumbar vertebra was selected because measuring cancellous bone-rich lumbar vertebra is appropriate to change in bone metabolism, for the ratio of the cancellous bone in lumbar vertebral bone is 80%, greater than in other bones, and a change in bone metabolism is more likely to occur in cancellous bone than cortical bone [13].

#### Image analysis and post-processing

Radiologist who was blinded to the clinical presentation and case control subjects examined collected images**.** The images were transferred to a workstation and were loaded into the DTI software provided by the vendor. Rectangular regions of interest (ROIs); avoiding the cortex; were set on the FA and ADC maps in the sagittal plane in L1-L4 lumbar vertebrae and in coronal plane in femoral neck on both sides. The mean ADC and FA values were computed.

#### Statistical analysis

Data were entered and analyzed using:

- IBM-SPSS software (IBM Corp. Released 2020. IBM SPSS Statistics for Windows, Version 27.0. Armonk, NY: IBM Corp).
- MedCalc® Statistical Software version 20 (MedCalc Software Ltd, Ostend, Belgium; https://www.medcalc.org; 2021).

Qualitative data were expressed as N (%). Quantitative data were initially tested for normality using Shapiro–Wilk’s test and were expressed as mean ± SD if normally distributed or median and interquartile range (Q1 or 25^th^ percentile – Q3 or 75^th^ percentile) if not.

#### Receiver operating characteristic (ROC) curve analysis

The Receiver Operating Characteristic (ROC) curve analysis was used to find a cutoff value of a continuous variable that can discriminate between two conditions.

## Results

The clinical and biochemical characteristics of the participants are presented in Table 1. There were no statistically significant differences in age, sex, or BMI between diabetic patients and control subjects.

**Table 1:** Characteristics of the study population

Parameter	Group A *N* = 30	Group B *N* = 38	Group C *N* = 30	*p*-value	Post hoc analysis		
					P1 A vs C	P2 B vs C	P3 A vs B
Female	24 (80%)	32 (84.2%)	25 (83.3%)	0.860			
Male	6 (20%)	6 (15.8%)	5 (16.7%)				
Age (years)	42.7 ± 5.4	44.9 ± 4.2	42.8 ± 4.8	0.104			
BMI (kg/m^2^)	33.9 ± 3.7	33.9 ± 4.8	33.9 ± 4.3	0.999			
HbA1c (%)	8.7 ± 2.1	10 ± 1.9		0.012			
DM duration (years)	5.5 ± 2.6	9.9 ± 4.1		< 0.001			
Creatinine (mg/dL)	1.05 ± 0.28	1.24 ± 0.3	0.78 ± 0.19	0.09			
25-OH vitamin D (ng/ml)	25.43 ± 11.84	25.83 ± 7.94	28.83 ± 8.94	0.071			
PTH (pg/ml)*	44.5 (38.5–48.3)	35 (25–38.3)	50 (45–52.8)	< 0.001	0.391	< 0.001	< 0.004
CTX (ng/l)*	17.7 (14.5–20.6)	16.6 (11.9–19.9)	21.7 (19.5–26)	< 0.001	0.012	< 0.001	0.539
P1NP (ng/ml)*	350 (311–398)	340 (288–379)	334 (309–391)	0.767			

Data are presented as mean ± SD or median (Q1-Q3)*

Group A: diabetic patients without microvascular complications

Group B: diabetic patients with microvascular complications

Group C: control subjects

*BMI* Body mass index, *PTH* Parathyroid hormone, *CTX* C-terminal cross linking telopeptide of type 1 collagen, *P1NP* Procollagen 1 N-terminal propeptide

The mean duration of diabetes and HbA1c levels were 9.9 ± 4.1 years and 10 ± 1.9% in diabetics with microvascular complications, compared to 5.5 ± 2.6 years and 8.7 ± 2.1% in those without complications (*p* < 0.001 and *p* = 0.012, respectively). Patients with complications had significantly lower PTH levels compared to both patients without complications and control subjects (*p* < 0.004 and *p* < 0.001, respectively).

Analysis of serum BTMs revealed that CTX levels were significantly decreased in both diabetic groups compared with controls (*p* = 0.012 and *p* < 0.001), but no significant difference was observed between the two diabetic groups (*p* = 0.539). P1NP levels did not differ significantly among the groups.

DEXA scan and MRI results are shown in Table 2. No statistically significant differences were found in BMD measurements among the three groups.

**Table 2:** Bone mineral density and DTI results of the studied groups

Parameter	Group A *N* = 30	Group B *N* = 38	Group C *N* = 30	*p*-value	Post hoc analysis		
					P1 A vs C	P2 B vs C	P3 A vs B
Lumbar spine aBMD (g/cm^2^)	1.18 (1.14–1.32)	1.19 (1.13–1.28)	1.23 (1.14–1.28)	0.565			
Femoral neck aBMD (g/cm^2^)	1.08 (1.03–1.1)	1.03 (0.96–1.09)	1.06 (0.94–1.13)	0.423			
lumbar spine t-score	-0.15 (-0.55 to 1.08)	-0.05 (-0.6 to 0.72)	0.25 (-0.13—0.53)	0.345			
Femoral neck t-score	0.75 (0.23 to 1)	0.35 (-0.2 to 1.3)	0.20 (-0.3 to 0.83)	0.222			
lumbar spine z-score	-0.4 (-1.1 to 0.08)	-0.35(-1.25 to 0.13)	-0.2 (-0.6 to 0.2)	0.300			
Femoral neck z-score	0.2 (-0.05 to 0.5)	-0.1 (-0.5 to 0.6)	-0.35 (-0.7 to 0.3)	0.101			
FA (spine)	0.694 (0.587-0.798)	0.648 (0.530-0.756)	0.786 (0.755-0.834)	< 0.001	0.018	< 0.001	1
FA (hip)	0.615 (0.469-0.743)	0.566 (0.462-0.626)	0.731 (0.636-0.827)	< 0.001	0.007	< 0.001	0.550
ADC (spine) × 10^–3^ mm^2^/s	0.421 (0.326-0.480)	0.458 (0.314-0.584)	0.316 (0.281-0.424)	0.004	0.184	0.003	0.973
ADC (hip) × 10^–3^ mm^2^/s	0.506 (0.443-0.582)	0.562 (0.504-0.635)	0.430 (0.356-0.500)	< 0.001	0.049	< 0.001	0.344

Data are presented as median (Q1-Q3)

Group A: diabetic patients without microvascular complications

Group B: diabetic patients with microvascular complications

Group C: control subjects

*aBMD* areal bone mineral density, *FA* Fractional anisotropy, *ADC* Apparent diffusion coefficient

There were statistically significant differences in all DTI measurements between the groups. Pairwise comparisons with Bonferroni correction revealed that FA of the spine (*p* = 0.018 and *p* < 0.001) and hip (*p* = 0.007 and *p* < 0.001) was significantly lower, while ADC of the hip was significantly higher in both diabetic groups compared to controls (*p* = 0.049 and *p* < 0.001). ADC of the spine was significantly higher in diabetic patients with complications compared to control subjects (*p* = 0.003).

Correlations between DTI parameters and other clinical and biochemical variables are presented in Tables 3 & 4.

**Table 3:** Correlation of FA values with studied parameters in diabetic patients and control group

	DM without complications				DM with complications				Control group			
	Spine		Hip		Spine		Hip		Spine		Hip	
	r	p	r	p	r	p	r	p	r	p	r	p
Age (years)	-0.213	0.368	-0.338	0.144	0.047	0.780	0.214	0.197	0.254	0.175	0.254	0.175
Male Sex	0.166	0.485	0.311	0.181	0.230	0.166	0.332	0.156	0.161	0.493	0.064	0.735
BMI (kg\m2)	0.052	0.755	-0.042	0.802	0.506	0.023	0.451	0.046	0.017	0.927	0.006	0.975
DM duration (years)	0.099	0.677	0.001	0.997	-0.013	0.939	0.208	0.210	-	-	-	-
HbA1c (⁒)	0.140	0.557	0.155	0.513	-0.103	0.538	-0.061	0.715	-	-	-	-
FA (spine)	1.00	-	0.447	0.048	1.00	-	0.551	< 0.001	1.00	-	0.756	< 0.001
FA (hip)	0.447	0.048	1.00	-	0.551	< 0.001	1	-	0.756	< 0.001	1.00	-
Mean ADC (spine)	-0.865	0.000	-0.629	0.003	-0.865	< 0.001	-0.518	< 0.001	-0.649	< 0.001	-0.622	< 0.001
Mean ADC (hip)	-0.319	0.171	-0.827	< 0.001	-0.359	0.027	-0.668	< 0.001	-0.275	0.167	-0.718	< 0.001
Lumbar spine aBMD (g/cm^2^)	0.163	0.492	-0.269	0.252	-0.226	0.173	-0.027	0.873	0.055	0.773	0.048	0.8
Femoral neck aBMD (g/cm2)	0.469	0.037	0.316	0.175	-0.071	0.670	0.075	0.657	0.077	0.685	0.255	0.173
PTH level (pg/ml)	-0.049	0.837	-0.180	0.448	0.458	0.004	0.336	0.039	-0.066	0.725	0.004	0.985
CTX level (ng/l)	0.098	0.681	0.008	0.972	-0.193	0.247	0.322	0.048	0.065	0.743	0.049	0.81
P1NP (ng/ml)	0.186	0.433	0.117	0.622	0.008	0.960	0.133	0.427	0.271	0.147	0.352	0.056

*BMI* Body mass index, *aBMD* areal bone mineral density, *FA* fractional anisotropy, *ADC* apparent diffusion coefficient, *PTH* parathyroid hormone, *CTX* C-terminal cross linking telopeptide of type 1 collagen, *P1NP* Procollagen 1 N-terminal propeptide

**Table 4:** Correlation of Mean ADC with studied parameters in diabetic patients and control group

	DM without complications				DM with complications				Control group			
	Spine		Hip		Spine		Hip		Spine		Hip	
	r	p	r	p	r	p	r	p	r	p	r	p
Age (years)	0.396	0.084	0.387	0.091	0.214	0.197	0.003	0.987	-0.295	0.114	-0.189	0.318
Male Sex	0.166	0.485	0.311	0.181	-0.112	0.503	-0.293	0.116	-0.339	0.067	-0.291	0.118
BMI (kg\m^2^)	-0.495	0.026	-0.441	0.052	0.216	0.192	-0.164	0.325	-0.126	0.506	-0.192	0.310
DM duration (years)	0.115	0.630	0.228	0.333	-0.008	0.961	-0.183	0.271	-	-	-	-
HbA1c (⁒)	-0.224	0.343	0.014	0.952	0.003	0.987	-0.218	0.188	-	-	-	-
Mean FA (spine)	-0.865	< 0.001	-0.319	0.171	-0.865	< 0.001	-0.359	0.027	-0.649	< 0.001	-0.275	0.167
Mean FA (hip)	-0.629	0.003	-0.827	< 0.001	-0.518	0.001	-0.668	< 0.001	-0.622	< 0.001	-0.718	< 0.001
Mean ADC (spine)	1.00	-	0.468	0.038	1.00	-	0.383	0.018	1.00	-	0.679	< 0.001
Mean ADC (hip)	0.468	0.038	1.00	-	0.383	0.018	1.00	-	0.679	< 0.001	1.00	-
Lumbar spine aBMD (g/cm^2^)	0.101	0.672	0.198	0.403	0.177	0.288	-0.029	0.863	-0.352	0.056	-0.265	0.157
Femoral neck aBMD (g/cm^2^)	-0.448	0.047	-0.375	0.103	0.022	0.894	-0.051	0.763	-0.085	0.655	0.073	0.702
PTH level (pg/ml)	-0.013	0.957	0.037	0.877	-0.418	0.009	-0.010	0.950	-0.010	0.960	-0.212	0.260
CTX level (ng/l)	-0.072	0.764	0.075	0.752	0.173	0.300	0.287	0.080	-0.270	0.149	-0.209	0.268
P1NP(ng/ml)	-0.015	0.950	-0.233	0.324	-0.120	0.473	0.029	0.865	-0.170	0.169	-0.312	0.093

*BMI* Body mass index, *aBMD* areal bone mineral density, *FA* fractional anisotropy, *ADC* apparent diffusion coefficient, *PTH* parathyroid hormone, *CTX* C-terminal cross linking telopeptide of type 1 collagen, *P1NP* Procollagen 1 N-terminal propeptide

FA of the spine was positively correlated with FA of the hip and negatively correlated with ADC of the spine, mean ADC of the hip showed a positive correlation with ADC of the spine in both groups, and a negative correlation with FA of the hip in both diabetic groups and control group. Additionally, ADC of the hip was negatively correlated with FA of the spine in patients with complicati\ons.

**Fig. 1:**
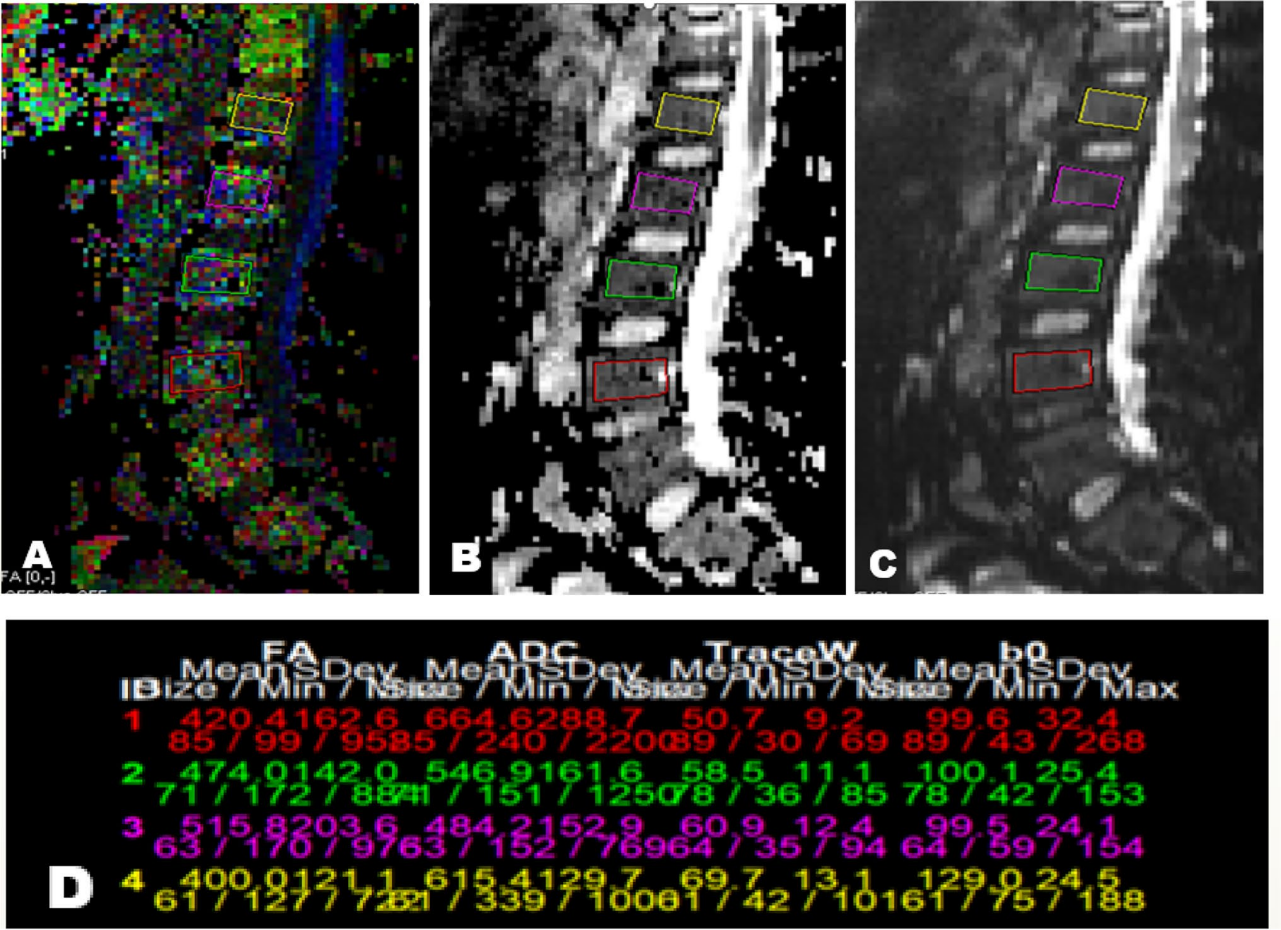
DTI of lumbosacral spine in sagittal plane of diabetic patient with microvascular complications, rectangular ROIs placed from L1-L4 vertebrae: **A**) Colored FA map, **B**) ADC map, **C**) b0 image, **D**) Corresponding FA & ADC values with mean FA value 0.452 and mean ADC value 0.577 × 10^–3^ mm^2^/s

**Fig. 2:**
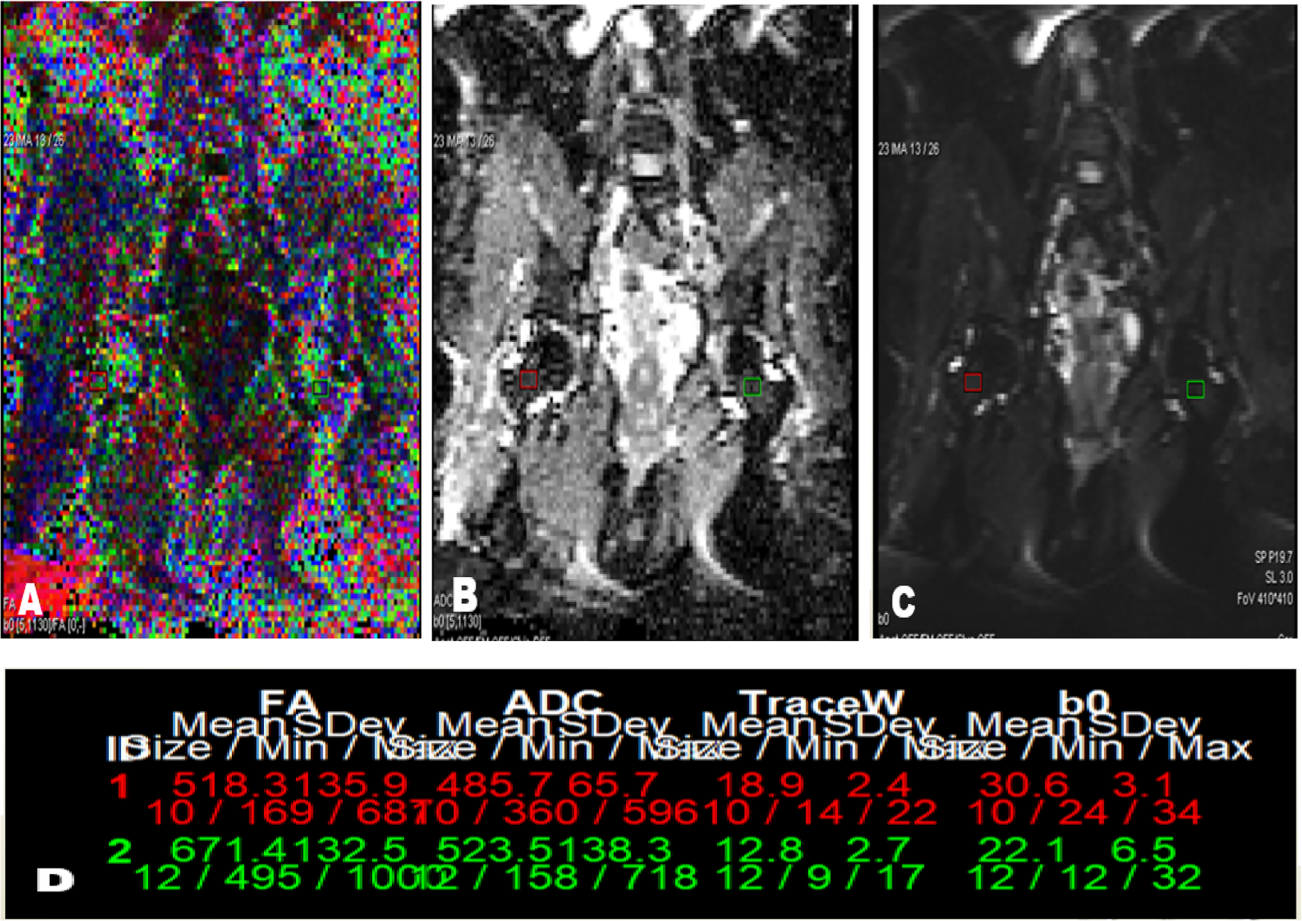
DTI of both hip joints in coronal plane of diabetic patient with microvascular complications with ROIs placed in femoral neck on both sides: **A**) Colored FA map, **B**) ADC map, **C**) b0 image, **D**) Corresponding FA and ADC values with mean FA value 0.594 and mean ADC value 0.504 × 10^–3^ mm^2^/s

In the group with microvascular complications, PTH level was positively correlated with FA of spine and hip and negatively correlated with ADC of spine. CTX level was positively correlated with FA of hip (Figs. 1 and 2).

**Fig. 3:**
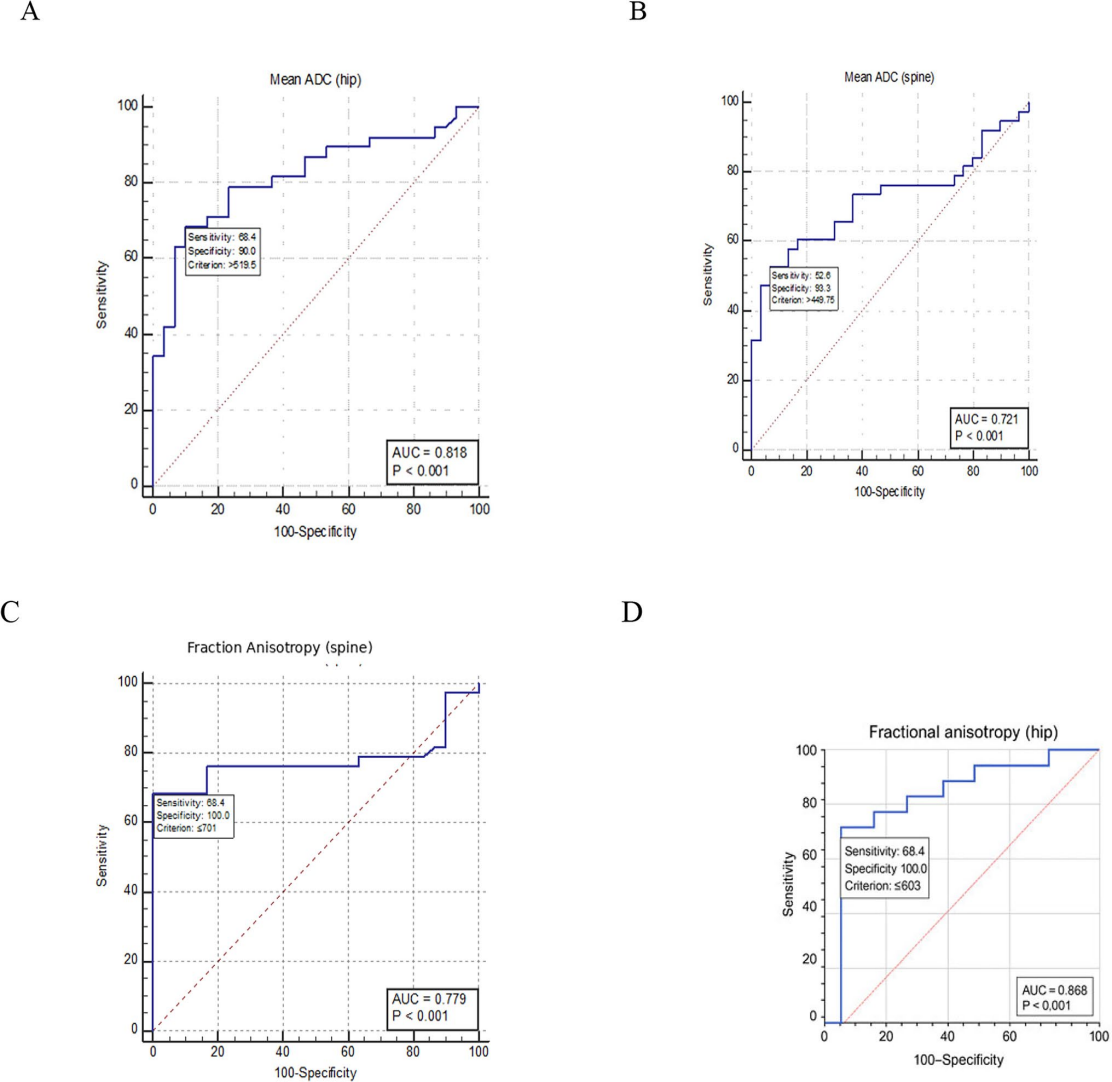
ROC curve analysis: Area under the curve (AUC), sensitivity, specificity, *p* -values and cut off values to discriminate all diabetic patients (N = 68) from control subjects (N = 30) using diffusion tensor imaging parameters; apparent diffusion coefficient (ADC) and fractional anisotropy for hip region (**A** & **D**) and spins (**B** & **C**)

ROC curve analysis done to discriminate all diabetic patients (*N* = 68) from control subjects (Table 5 & Fig. 3) and revealed that FA and ADC values of the hip had the highest area under the curve (AUC) values—0.868 and 0.818, respectively—followed by FA and ADC of the spine (0.779 and 0.721, respectively). The optimal cut-off values providing the best sensitivity and specificity were observed for FA measurements, which outperformed ADC in diagnostic performance.

**Table 5:** ADC, FA and CTX cutoff values to discriminate all Diabetic patients from control group(based on ROC curve analysis)

Parameter	Cutoff	AUC	p-value	Sensitivity	Specificity
ADC (hip)	> 0.520	0.818	< 0.001	68.4	90
ADC (spine)	> 0.450	0.721	< 0.001	52.6	93.3
FA (hip)	≤ 0.603	0.868	< 0.001	68.4	100
FA (spine)	≤ 0.701	0.779	< 0.001	68.4	100
CTX	≤ 17.3	0.835	< 0.001	60.5	96.7

*AUC* Area under the curve, *CTX* C-terminal cross linking telopeptide of type 1 collagen

*FA* Fractional anisotropy, *ADC* Apparent diffusion coefficient

## Discussion

Along with the more frequent complications of nephropathy, heart disease, and diabetic retinopathy, DM can harm the skeletal system, leading to bone loss and even osteoporosis [14]. In the present study, no difference was found in BMD between diabetics and nondiabetics. According to the findings of various research studies, BMD may decrease, remain unchanged, or even increase in people with T2DM [15].

DXA has limited ability to predict a patient's risk of bone fracture.This lack of sensitivity is probably caused by the incomplete information that BMD provides on the characteristics of cancellous bone, evaluating just its mineral component. In this context, MRI techniques permit evaluation of the physiological and functional changes that occur with diabetes [16].

FA is the primary indicator of a tissue's anisotropy. Its value ranges from 0 for perfect isotropy to 1 for complete anisotropy. FA measures how much a tissue's organization impacts the direction of water molecule [17]. If a well-organized cellular architecture has been disrupted by a physiologic or pathologic process this means that a highly anisotropic tissue becomes less anisotropic (FA decreases) [18]. Our study showed a statistically significant decrease in FA measured for both vertebral spines and hip region, in diabetic patients with and without microvascular complications compared with control subjects, but not between the two diabetic groups, which reflects an early disrupted cellular architecture of bone in diabetic patients.

The ADC reflects the degree of freedom of water molecule motion. As the motion becomes more fluid, the ADC increases. In other words, ADC and mean diffusivity represent the extent to which water molecule movement is hindered by barriers, whether these are physiological or induced by pathological processes [19]. Altered bone microstructure appears to lead to increased porosity, which in turn elevates ADC values [20].

In this study, ADC of the hip was significantly higher in both diabetic groups compared to the control group, indicating early involvement of the hip and suggesting an increased risk of hip fractures relative to other fracture types in diabetic patients [21]. Additionally, ADC of the spine was significantly higher in the diabetic group with microvascular complications compared to controls, suggesting that the elevation in spinal ADC may be part of diabetic microangiopathy affecting the bone marrow's small vessels—a condition referred to as diabetic osteopathy. Prolonged hyperglycemia increases the accumulation of advanced glycation end-products (AGEs) in bone collagen, which negatively impacts the material and biomechanical properties of cortical bone [22].

In our study, diabetic patients had a significantly lower PTH level than the control group. This finding agrees with previous studies that found lower PTH levels in T2DM patients than non diabetics [23, 24]. A subtle hypoparathyroidism could lead to a low bone turnover state that may contribute to the bone fragility in DM patients [23].

In addition, bone turnover can be assessed using biochemical markers. These markers are produced during the dynamic process of bone remodeling, reflecting bone metabolism over short periods and offering better prediction of recent changes [25]. CTX is a degradation product of mature type I collagen, while P1NP is a product of type I collagen formation by osteoblasts [26]. Both have been recommended by the International Osteoporosis Foundation as the preferred markers for evaluating bone resorption (CTX) and bone formation (P1NP) in both clinical and research settings [27].

In our study, diabetic patients—with and without microvascular complications—showed a significant decrease in CTX levels compared to the control group, while P1NP levels did not differ significantly. Although P1NP levels were lower in the group with microvascular complications compared to the other two groups, the difference did not reach statistical significance. Previous studies have also reported that both formation and resorption BTMs are decreased in diabetic patients, supporting the notion that diabetes mellitus represents a state of low bone turnover, which may contribute to increased fracture risk [5, 28].

According to the current study, the low bone turnover state observed in diabetic patients—evidenced by reduced PTH and CTX levels—was significantly correlated with both FA and ADC values. This suggests that the loss of bone organization, indicated by decreased FA, and the increase in bone porosity, reflected by elevated ADC, are associated with impaired bone remodeling. This low turnover state may lead to the accumulation of microcracks, deterioration of bone quality, and structural disorganization [29].

Among diabetic patients with microvascular complications, a positive correlation was found between mean FA values of the spine and hip and body mass index (BMI). Although obesity has been shown in many studies to be a protective factor against osteopenia and osteoporosis in the spine and femur [30–32], the presence of anisotropic bone tissue in our diabetic patients suggests that their bone may exhibit different mechanical behaviors depending on the direction of the applied load [33].

Although the parameters measured by DTI in our study did not demonstrate high sensitivity for distinguishing between diabetic patients and controls, they did show good specificity. The highest specificity—above 90%—was observed for spine and hip FA, as well as spine ADC, with AUC values of 0.868, 0.779, and 0.721, respectively.

To our knowledge, this is the first study to apply DTI for the assessment of bone microarchitecture in diabetic patients. Nevertheless, the study had several limitations. First, the cutoff values should be interpreted with caution due to the limited sample size. Second, we excluded patients with a history of fractures and those already receiving anti-resorptive or anabolic therapy; therefore, our findings may not be generalizable to diabetic individuals at the highest risk of fracture. These populations should be considered in future research.

## Conclusion

DTI parameters—FA and ADC of bone marrow water—can provide valuable insights into the microstructural changes associated with the development of a low bone turnover state in diabetes. This may help explain why individuals with type T2DM are at greater risk of fragility fractures, despite having normal or even higher average bone mineral density. This technique may have important implications for future clinical practice, enabling the early identification of high-risk patients—specifically those with lower FA and higher ADC values than the defined cut-off thresholds.

## Data Availability

The data are not publicly available due to patient privacy regulations but can be provided by the corresponding author upon reasonable request and institutional approval.
